# Clinical Characteristics and Outcomes of Patients With Mpox Who Received Tecovirimat in a New York City Health System

**DOI:** 10.1093/ofid/ofad552

**Published:** 2023-11-02

**Authors:** Christopher Vo, Rustin Zomorodi, Richard Silvera, Logan Bartram, Luz Amarilis Lugo, Erna Kojic, Antonio Urbina, Judith Aberg, Keith Sigel, Rachel Chasan, Gopi Patel

**Affiliations:** Division of Infectious Diseases, Department of Medicine, Icahn School of Medicine at Mount Sinai, NewYork, New York, USA; Division of Infectious Diseases, Department of Medicine, Icahn School of Medicine at Mount Sinai, NewYork, New York, USA; Division of Infectious Diseases, Department of Medicine, Icahn School of Medicine at Mount Sinai, NewYork, New York, USA; Division of Infectious Diseases, Department of Medicine, Icahn School of Medicine at Mount Sinai, NewYork, New York, USA; Division of Infectious Diseases, Department of Medicine, Icahn School of Medicine at Mount Sinai, NewYork, New York, USA; Division of Infectious Diseases, Department of Medicine, Landspítali University Hospital, Reykjavík, Iceland; Division of Infectious Diseases, Department of Medicine, Icahn School of Medicine at Mount Sinai, NewYork, New York, USA; Division of Infectious Diseases, Department of Medicine, Icahn School of Medicine at Mount Sinai, NewYork, New York, USA; Division of Infectious Diseases, Department of Medicine, Icahn School of Medicine at Mount Sinai, NewYork, New York, USA; Division of Infectious Diseases, Department of Medicine, Icahn School of Medicine at Mount Sinai, NewYork, New York, USA; Division of Infectious Diseases, Department of Medicine, Icahn School of Medicine at Mount Sinai, NewYork, New York, USA

**Keywords:** HIV/AIDS, mpox, STI, tecovirimat

## Abstract

**Background:**

The 2022 global mpox outbreak was notable for transmission between persons outside of travel and zoonotic exposures and primarily through intimate contact. An understanding of the presentation of mpox in people with human immunodeficiency virus (HIV) and other immunocompromising conditions and knowledge of the efficacy of tecovirimat continue to evolve.

**Methods:**

This retrospective study describes clinical features and outcomes of persons with mpox who received tecovirimat. Data were obtained via medical record review of patients prescribed tecovirimat in a health system in New York City during the height of the outbreak in 2022.

**Results:**

One hundred thirty people received tecovirimat between 1 July and 1 October 2022. People with HIV (n = 80) experienced similar rates of recovery, bacterial superinfections, and hospitalization compared to patients without immunocompromising conditions. Individuals determined to be severely immunocompromised (n = 14) had a higher risk of hospitalization than those without severe immunocompromise (cohort inclusive of those with well-controlled HIV, excluding those without virologic suppression, n = 101): 50% versus 9% (*P* < .001). Hospitalized patients (n = 18 [13% of total]) were primarily admitted for bacterial superinfections (44.4%), with a median hospital stay of 4 days. Of those who completed follow-up (n = 85 [66%]), 97% had recovery of lesions at time of posttreatment assessment. Tecovirimat was well tolerated; there were no reported severe adverse events attributed to therapy.

**Conclusions:**

There were no significant differences in outcomes between people with HIV when evaluated as a whole and patients without immunocompromising conditions. However, mpox infection was associated with higher rates of hospitalization in those with severe immunocompromise, including patients with HIV/AIDS. Treatment with tecovirimat was well tolerated.

Key Points: In our mpox cohort, people with HIV had similar rates of recovery and complications as those without HIV or other immunocompromising conditions. Severe immunocompromise was associated with a higher hospitalization rate. Tecovirimat was well tolerated, with minimal side effects.

## Teaser

In this retrospective study of patients with mpox who received tecovirimat in New York City during the 2022 outbreak, HIV status alone did not significantly impact clinical outcomes. However, severe immunocompromise was associated with a higher hospitalization rate.

Mpox is a zoonotic virus in the *Orthopoxvirus* genus of the Poxviridae family, first reported in humans in Central Africa in 1970 [1, 2]. Sporadic outbreaks have occurred since [3–5]. Human-to-human transmission has been well documented, with few cases occurring beyond Africa or without animal exposure prior to recent events [1, 5–8].

Widespread person-to-person transmission of mpox in 2022 prompted the World Health Organization (WHO) to declare mpox a global public health emergency in July 2022 [9]. Since the recognition of a non-travel-related mpox case in the United Kingdom in May 2022, there have been >89 000 cases of mpox reported in >100 countries, and >150 associated deaths [10]. In the United States, as of August 2023, approximately 30 600 mpox cases and 46 deaths have been reported by the Centers for Disease Control and Prevention (CDC) [11]. This outbreak was unique in that that it has disproportionately affected men identifying as gay and/or bisexual and other men who have sex with men (MSM), as well as transgender and gender-diverse adults [6, 12–15].

The 2022 human mpox outbreak is attributed to clade IIb, which has demonstrated self-limited and milder disease compared to clades I and IIa, which are endemic to parts of Africa [1, 7, 16, 17]. Although our understanding of this outbreak and its clinical impact on specific populations continues to evolve, most patients have experienced rash, often with genital and mucosal involvement. However, pain associated with mucosal disease has been described in cases as severe enough to impede bowel function and/or oral intake [7, 18–20].

A recent study demonstrated that people with human immunodeficiency virus infection (PWH) who received tecovirimat had similar rates of hospitalization, concurrent infections, and overall treatment outcomes compared to patients without human immunodeficiency virus (HIV) [15]. However, significantly immunocompromised persons, including those with advanced HIV infection, appeared to be at increased risk for severe outcomes and complications, including death [7, 13, 18].

Given the broad range in presentations in the 2022 mpox outbreak and the increasing recognition of severe disease in vulnerable patients, there is a clear need for effective therapy. Despite previous outbreaks, there is currently no commercially available treatment for human mpox. Tecovirimat, an antiviral agent targeting the orthopoxvirus p37 protein, is licensed for treatment of smallpox, and became available in June 2022 for clinical use in the United States under an expanded-access investigational new drug (EA-IND) protocol for the treatment of human mpox [1, 13, 21]. In vitro testing demonstrated activity against both smallpox and mpox, and tecovirimat was found to be well tolerated by healthy human volunteers [22–30]. There are small case series and retrospective observational studies that have since demonstrated safety in patients with mpox [14, 15, 18–20, 31–34]. Currently, there is an ongoing clinical trial, Study of Tecovirimat for Human Mpox Virus (STOMP), funded by the National Institute of Allergy and Infectious Diseases, which aims to systematically study the clinical efficacy of tecovirimat for treatment of mpox.

In this retrospective cohort study, we describe clinical outcomes of 130 patients diagnosed with mpox during the 2022 outbreak who were prescribed tecovirimat through the CDC EA-IND protocol.

## METHODS

### Study Design and Participants

We reviewed the demographic and clinical characteristics of individuals prescribed tecovirimat at clinical sites within the Mount Sinai Health System for suspected or confirmed mpox infection between 1 July and 1 October 2022. All included patients were clinically assessed and consented to being prescribed tecovirimat through the CDC EA-IND protocol [13].

Per protocol, both intravenous (IV) and oral tecovirimat were prescribed for patients with suspected or confirmed mpox if deemed appropriate by the evaluating infectious diseases clinician. Informed consent for treatment with tecovirimat was performed in accordance with the CDC protocol, which was also approved by the Icahn School of Medicine at Mount Sinai (ISMMS) Institutional Review Board (IRB). This retrospective study was approved by the ISMMS IRB under a different protocol.

### Microbiologic Diagnostic Testing

Suspected or confirmed mpox cases were defined as individuals with either laboratory-confirmed infection or a high index of suspicion based on clinical history, risk factors, and physical examination. Unless testing was deemed unnecessary by the treating clinician, suspected cases were tested using either a pan-orthopoxvirus or mpox virus reverse-transcription polymerase chain reaction (RT-PCR) assay performed at the Mount Sinai Clinical Microbiology Laboratory or by commercial or public health laboratories.

### Data Collection and Assessment on Tecovirimat Therapy

We extracted demographic information, clinical data and outcomes, data on treatment, and laboratory results including orthopoxvirus- or mpox-specific RT-PCR results from CDC EA-IND case report forms and electronic medical records.

As part of the initial treatment protocol, patients were assessed at baseline, while on the 14-day tecovirimat treatment course (on-treatment), and within 3–14 days after completion of therapy. However, following an amendment to the protocol on 20 July 2022, only 1 follow-up visit was recommended either during or after treatment. Standardized assessments were provided as part of the protocol, although assessments were adjusted throughout the course of this study based on modifications made to the CDC protocol [22]. Testing for other sexually transmitted infections (STIs) including 3-site testing for gonorrhea and chlamydia, were recommended (but not required) as part of institutional guidance.

We obtained the following data for each patient at initial presentation: age; gender; patient-reported race and ethnic group; history of smallpox or mpox vaccination; medical comorbidities; sexual history; HIV history including CD4 cell count, viral load, and antiretroviral therapy (ART) regimen; history of immunocompromise (per the CDC guidance on tecovirimat, defined as advanced or poorly controlled HIV [CD4 count <200 cells/μL and/or ≤13% total lymphocyte count with viral load >200 copies/mL], leukemia, lymphoma, generalized malignancy, solid organ transplantation, or therapy with alkylating agents, antimetabolites, radiation, or tumor necrosis factor inhibitors, or high-dose corticosteroids) [21]; use of HIV preexposure prophylaxis (PrEP) therapy; presence of concomitant STI diagnoses; symptoms at presentation; date of rash onset, approximate number of lesions upon initiation of tecovirimat, and location of rash; and need for hospitalization. During follow-up and posttreatment assessments we collected the following data: recovery from orthopoxvirus infection with or without sequalae, time to clinical improvement and/or complete resolution, and side effects attributed to tecovirimat.

All patients with suspected or confirmed mpox virus were counseled on isolation precautions.

### Statistical Analysis

We summarized patient characteristics and outcomes using basic descriptive statistics and analysis. We then compared characteristics and outcomes for immunosuppressed versus nonimmunosuppressed patients using the χ^2^ test for binary/categorical factors and the Wilcoxon test for continuous variables. Statistical analysis was done by using Stata statistical software (version 16).

## RESULTS

### Baseline Demographics and Clinical Characteristics

A total of 130 patients were prescribed tecovirimat; 120 patients had laboratory-confirmed disease (92.3%), and 10 patients (7.7%) had suspected mpox based on clinical history and presentation and were not tested (Table 1).

**Table 1.** Demographic and Clinical Characteristics of Persons With Mpox

Characteristic	All Patients (N = 130)
Age, y, median (range)	37 (20–58)
Gender	
Male	124 (95.4)
Female	1 (0.8)
Nonbinary	3 (2.3)
Transgender woman	1 (0.8)
Unknown or not reported	1 (0.8)
Sexual behaviors	
MSM	121 (93)
Heterosexual	1 (0.8)
Sex with men and women	3 (2.3)
Other or unknown	5 (3.8)
Ethnicity	
Hispanic or Latinx	32 (24.6)
Not Hispanic or Latinx	88 (67.7)
Unknown or not reported	10 (7.7)
Race	
White	62 (47.7)
Black	46 (35.4)
Asian	9 (6.9)
Other or unknown	13 (10)
HIV positive	80 (61.5)
Use of PrEP against HIV, no./No. (%)	40/50 (80)
Other medical conditions	
Atopic dermatitis or eczema–active	7 (5.4)
Autoimmune/connective tissue disorder	7 (5.4)
Other skin disease	4 (3.1)
Hematologic stem cell or solid organ transplant	3 (2.3)
Lymphoma or lymphoproliferative disorder	1 (0.8)
Other cancer	4 (3.1)
Severely immunocompromised	14 (10.7)
HIV/AIDS (CD4 <200 cells/μL) with detectable VL (>200 copies/mL)	7 (50)
Other immunosuppressive conditions	
IBD (including ulcerative colitis or Crohn disease on immunosuppressants)	3 (21.4)
Kidney transplant	2 (15.3)
HIV/AIDS and multicentric Castleman disease	1 (7.1)
Systemic lupus erythematosus	1 (7.1)
Reported history of at least 1 vaccinia vaccination	41 (31.5)
Concurrent diagnosis of superimposed bacterial infection	19 (14.6)
Epiglottitis	1
Pharyngitis	3
Pharyngitis and proctitis	1
Penile cellulitis	5
Periorbital cellulitis	1
Peritonsillar abscess	1
Proctitis	1
Superimposed cellulitis	5
Superimposed cellulitis with bacteremia	1
New concurrent diagnosis of chlamydia	13 (10)
New concurrent diagnosis of gonorrhea	17 (13.1)
New concurrent diagnosis of syphilis	11 (8.5)

Data are presented as No. (%) unless otherwise indicated. Surveyed and reported characteristics of the entire cohort included basic demographics, presence of other medical comorbidities and immunocompromising conditions, use of PrEP, history of vaccinia vaccination, and presence of superimposed bacterial infections. Diagnoses of gonorrhea and chlamydia included pharyngeal, urethral, and rectal involvement.

Abbreviations: HIV, human immunodeficiency virus; IBD, inflammatory bowel disease; MSM, men who have sex with men; PrEP, preexposure prophylaxis.

The median patient age was 37 years. Most of this cohort identified as male (95.4%) and 4 (3.1%) individuals identified as nonbinary or transgender. One hundred twenty-one (93%) patients’ sexual practices were described as MSM, 3 (2.3%) as men who have sex with men and women, and 1 patient (0.8%) as heterosexual. Thirty-two (24.6%) identified as Hispanic or Latinx, 62 (48%) as White, 46 (35%) as Black, and 9 (7%) as Asian, and 13 (10%) reported race as other or unknown/not reported. A severe immunocompromising condition was present in 14 patients (10.7%). Forty-one (31.5%) individuals reported receiving at least 1 dose of a vaccinia vaccine (primarily live, nonreplicating smallpox and mpox vaccine) prior to developing mpox infection. Forty patients reported receiving at least 1 dose of the smallpox and mpox live, nonreplicating vaccine (JYNNEOS). Twenty-two received at least 1 dose a median of 8 days (range, 1–40 days) prior to onset of illness. Seven reported at least 1 dose prior to onset of illness, but date of administration was unknown and 11 received at least 1 dose on the day of or a median of 3 days after onset of illness (range, 0–7 days). Only 1 patient reported receiving ACAM2000 45 years prior to presentation.

A total of 80 (61.5%) of the patients in this cohort had HIV, of which the majority (93.8%) reported adherence with ART or laboratory-confirmed undetectable HIV viral load at time of mpox infection. Nearly a fifth of patients did not have HIV virologic suppression (19%), 14% had CD4 counts <200 cells/μL, and 16% met criteria for AIDS (Supplementary Table 1). Of note, PrEP was prescribed to 80% of persons who were not known to have HIV infection (n = 40).

### Clinical Findings

The clinical characteristics of mpox in this cohort are summarized in Table 2. The majority of patients (99.2%) had visible, characteristic skin lesions (Supplementary Figure 1), while 1 patient presented without visible lesions but with symptoms of proctitis. Most patients (>80%) also presented with at least 1 sign or symptom of systemic illness. Many had anogenital involvement. Mucosal involvement occurred in approximately a fifth of patients (21.5%), and ocular infection occurred in a minority of patients (n = 5 [3.8%]). The median time from illness onset to first appointment/evaluation for tecovirimat was 8 days.

**Table 2.** Diagnosis and Clinical Characteristics of Mpox

Characteristic	All Patients (N = 130)
Medical setting of presentation	
Outpatient	112 (86.2)
Inpatient	18 (13.8)
Laboratory confirmation of mpox	119 (91.5)
Signs and symptoms^a^	
Skin rash	129 (99.2)
Pain	99 (76.2)
Pain with defecation due to lesions near or on anus	73 (56.1)
Fever and/or chills	64 (49.2)
Lymphadenopathy	45 (34.6)
Pain with swallowing or sore throat	45 (34.6)
Myalgia	33 (25.4)
Fatigue	33 (25.4)
Initial No. of skin lesions	
0–10	37 (28.5)
11–100	86 (66.2)
>100	5 (3.8)
No lesions or missing data	2 (1.5)
Size of maximal lesion, mm, median (range)	5 (1–38)
Percentage of body affected, %, median (range)	10 (1–100)
Area of rash distribution per major anatomical area	
Skin	115 (88.5)
Anogenital	92 (70.8)
Mucosal	28 (21.5)
Ocular	5 (3.8)

Data are presented as No. (%) unless otherwise indicated. Baseline diagnoses and clinical characteristics documented in initial evaluation for tecovirimat.

^a^Signs/symptoms in less than 25% included headache, dysuria, hematochezia, night sweats, nausea/vomiting, dysphagia, abdominal pain, and genital swelling.

Concurrent STIs were common: 13 (10%) patients were found to have chlamydia (irrespective of anatomical site), 17 (13.1%) gonorrhea (irrespective of anatomical site), and 11 (8.5%) syphilis. Nineteen (14.6%) patients had concurrent or superimposed bacterial infections associated with their mpox lesions (9 patients had a microbiologic diagnosis; the rest were made clinically based on examination and imaging) (Table 1).

### Clinical Outcomes and Complications of Initial Cohort Prescribed Tecovirimat

Of the initial cohort of 130 patients prescribed tecovirimat, 85 patients (65.4%) were seen for follow-up posttreatment assessment and confirmed to have completed therapy. The primary reasons for initiating tecovirimat per the CDC EA-IND protocol were as follows: lesions in sensitive anatomical areas (83.1%), pain (67.7%), and risk of severe outcome due to immunosuppression (23.8%). The majority of patients (86.2%) initiated tecovirimat in the outpatient setting, while the remainder were hospitalized. Three (2.3%) patients required IV tecovirimat due to severe dysphagia. Four patients were confirmed to have never started the prescribed therapy and were excluded from outcome assessment. Five patients started treatment but did not complete the 14-day course. Reasons cited for incomplete treatment include leaving the clinical encounter prior to treatment initiation (n = 1), rapid symptom resolution (n = 3), and side effects (n = 1). Patients were seen in clinic for posttreatment assessment a median of 8 days after treatment completion.

The majority of patients, 81 of 85 (95.2%) with completed follow-up, reported complete recovery from mpox at the time of posttreatment assessment. A minority of patients (n = 4 [4.7%]) had persistence of symptoms at the time of posttreatment assessment. No patient deaths were observed within the timeframe of the study. The median time to improvement in lesions and/or pain on tecovirimat for all patients was 3 days (range, 1–12 days). The median time for complete lesion resolution on tecovirimat for all patients was 10 days (range, 3–20 days). PWH also experienced a high rate of clinical recovery. There were no serious or life-threatening adverse events recorded or attributed to tecovirimat. During the course of this study, 7 of 85 (8.2%) patients reported side effects (Supplementary Table 2).

### Clinical Outcomes and Complications by Subcohort

#### Patients With HIV

Eighty patients (61.5%) were PWH (Table 3; Supplementary Table 1). In comparison to patients without any immunocompromising conditions (n = 46), there was no significant difference between proportion of hospitalization (16.3% vs 10.9%; *P* = .41) or concurrent bacterial superinfection (12.5% vs 17.4%; *P* = .45). There was no significant difference in recovery between these groups (96.2% vs 93.5%; *P* = .58).

**Table 3.** Clinical Outcomes of Patients With Human Immunodeficiency Virus Versus Patients Without Immunocompromising Conditions

Characteristic	Immunocompetent Patients (n = 46)	PWH (n = 80)	*P* Value
Hospitalized	5 (10.9)	13 (16.3)	.41
No. of skin lesions			.32
0–10	12 (26.1)	24 (30)	
11–100	42 (91.3)	52 (65)	
>100	1 (2.2)	4 (5)	
Not reported	1 (2.2)	…	
Size of maximal lesion, mm, median (range)	4.5 (1–38)	4 (2–25)	.67
Percentage of body affected, %, median (range)	10 (1–80)	10 (1–100)	.25
Area of rash distribution per major anatomical area			
Skin	41 (89.1)	71 (88.8)	.95
Anogenital	30 (65.2)	58 (72.5)	.39
Oral mucosal	14 (30.4)	14 (17.5)	.09
Ocular	4 (8.7)	2 (2.5)	.51
Concurrent diagnosis of superimposed bacterial infection	8 (17.4)	10 (12.5)	.45
Cellulitis	1	3	
Cellulitis with bacteremia	0	1	
Epiglottitis	1	0	
Penile cellulitis	2	3	
Pharyngitis	1	2	
Pharyngitis and proctitis	1	0	
Periorbital cellulitis	1	0	
Peritonsillar abscess	0	1	
Proctitis	1	0	
Tonsillitis	0	0	
Drug formulation (oral vs IV), no./No. (%)	46/46 (100)	77/80 (96.3)	.18
Completed or documented clinical outcomes, no./No. (%)	31/46 (67.4)^a^	53/80 (66.3)^a^	.89
Completion of 14 d of therapy, no./No. (%)	28/31 (90.3)	47/53 (88.6)	.81
Clinical outcome, no./No. (%)			
Recovered from mpox infection	29/31 (93.5)	51/53 (96.2)	.58
Lesions or pain first started to improve, treatment day, median	4	3	.17
Lesions and pain fully resolved, treatment day, median	13.5	10	.18

Data are presented as No. (%) unless otherwise indicated. Posttreatment clinical outcomes of PWH compared to patients without human immunodeficiency virus or any other immunocompromising conditions.

Abbreviations: IV, intravenous; PWH, people with human immunodeficiency virus.

^a^Two patients never started therapy, so respective clinical outcomes are excluded.

#### Patients With Severe Immunocompromise

Our cohort had 14 patients who were severely immunocompromised (Table 4). In comparison to the rest of the cohort (including patients with well-controlled HIV but excluding PWH without virologic control, n = 101), patients with severe immunocompromising conditions had a higher proportion of hospitalizations (50% vs 9%; *P* < .001) and higher usage of IV tecovirimat (14.3% vs 0; *P* < .001). There was no significant difference in bacterial superinfections when compared to patients without severe immunocompromise (21.4% vs 13.8%; *P* = .46). Clinical outcomes were similar at time of posttreatment assessment; 85.7% of the severely immunocompromised patients with documented outcomes experienced clinical recovery compared with 97.1% of the rest of the cohort (*P* = .14).

**Table 4.** Clinical Outcomes of Patients Without Severe Immunocompromise Versus Severely Immunocompromised Patients

Characteristic	Immunocompetent Patients (n = 101; Excluding PWH Without Virologic Suppression)	Patients With Severely Immunocompromising Conditions (n = 14)	*P* Value
Hospitalized	9 (8.9)	7 (50)	**<**.**001**
No. of skin lesions			.42
0–10	28 (27.7)	5 (35.7)	
11–100	69 (68.3)	8 (57.1)	
>100	2 (2)	1 (7.1)	
Not reported	2 (2)	0	
Size of maximal lesion, mm, median (range)	5 (1–38)	5 (2–20)	.95
Percentage of body affected, %, median (range)	10 (1–100)	10 (2–90)	.59
Area of rash distribution per major anatomical area			
Skin	89 (88)	13 (92.8)	.66
Anogenital	72 (71.3)	7 (50)	.11
Oral mucosal	21 (20.8)	1 (7.1)	.22
Ocular	4 (4)	2 (14.3)	.10
Concurrent diagnosis of superimposed bacterial infection	14 (13.8)	3 (21.4)	.46
Cellulitis	3	0	
Cellulitis with bacteremia	0	1	
Epiglottitis	1	0	
Penile cellulitis	3	1	
Pharyngitis	3	0	
Pharyngitis and proctitis	1	0	
Periorbital cellulitis	1	0	
Peritonsillar abscess	0	1	
Proctitis	1	0	
Tonsillitis	1	0	
Drug formulation (oral vs IV), no./No. (%)	101/101 (100)	12/14 (85.3)	**<**.**001**
Completed or documented clinical outcomes, no./No. (%)	71/101 (70.3)^a^	7/14 (50)	.13
Completion of 14 d of therapy, no./No. (%)	67/70 (95.7)^b^	7/7 (100)	.58
Clinical outcome, no./No. (%)			
Recovered from mpox infection	68/70 (97.1)	6/7 (85.7)	.14
Lesions or pain first started to improve, treatment day, median	3	6.5	.17
Lesions and pain fully resolved, treatment day, median	11	12	.68

Data are presented as No. (%) unless otherwise indicated. Severely immunocompromised patients within this study are described based on the Centers for Disease Control and Prevention guidance on tecovirimat as having 1 of the following conditions: advanced or poorly controlled human immunodeficiency virus (CD4 count <200 cells/μL or ≤13% total lymphocytes with viral load >200 copies/mL); leukemia; lymphoma; generalized malignancy; solid organ transplantation; therapy with alkylating agents, antimetabolites, radiation, tumor necrosis factor inhibitors, or high-dose corticosteroids; being a recipient of a hematopoietic stem cell transplant <24 months posttransplant or ≥24 months but with graft-versus-host disease or disease relapse; or having autoimmune disease with immunodeficiency as a clinical component. PWH with uncontrolled viral load (>200 copies/mL) but with CD4 count >200 cells/mL or CD4 >13% total lymphocytes were not considered to be immunocompetent, as they had active viremia, and were thus excluded.

Abbreviations: IV, intravenous; PWH, people with human immunodeficiency virus.

^a^One patient never started therapy, so respective clinical outcomes are excluded.

^b^Patients self-discontinued treatment prior to the 14-day duration for the following reasons: 2 had complete symptom resolution prior to completion of the prescribed 14 days of therapy, so patients self-opted to have shortened course; 1 stopped due to side effects.

Bolded values indicate statistical significance.

#### Hospitalized Patients

There were 18 patients hospitalized during the study period, 3 (16.7%) of whom required care in an intensive care unit (Table 5). Many of these hospitalized patients were admitted for bacterial superinfection (n = 8 [44.4%]). The median duration of hospital stay was 4 days. Most of these patients were PWH (72.2%) and were not virally suppressed (61.5%). Only 8 of 18 (44.4%) were evaluated at their posttreatment appointment (the rest were lost to follow-up). Of these patients, 87.5% (n = 7) reported recovery by time of their posttreatment assessment. One patient remained hospitalized due to evolving disease and bacterial superinfection at the end of the study follow-up period.

**Table 5.** Diagnosis, Clinical Characteristics, and Clinical Outcomes of Hospitalized Patients

Characteristic	Hospitalized Patients (n = 18)
Medical setting of presentation	
Wards	15 (83.3)
Intensive care unit	3 (16.7)
No. of skin lesions	
0–10	8 (44.4)
11–100	9 (50)
>100	0
Not reported	1 (5.6)
Size of maximal lesion, mm, median (range)	4 (2–38)
Percentage of body affected, %, median (range)	10 (2–80)
Area of rash distribution per major anatomical area	
Skin	18 (100)
Anogenital	11 (61.1)
Oral mucosal	2 (11.1)
Ocular	3 (16.7)
Concurrent diagnosis of superimposed bacterial infection	8 (44.4)
Epiglottitis	0
Penile cellulitis	3
Periorbital cellulitis	1
Peritonsillar abscess	1
Pharyngitis and proctitis	0
Pharyngitis	0
Proctitis	1
Superimposed cellulitis	2
Duration of hospital stay, d, median	4
Reported history of at least 1 smallpox vaccination	1 (5.6)
HIV positive	13 (72.2)
Immunocompromising conditions	7 (38.9)
HIV/AIDS	4
IBD on immunosuppressants	1
Kidney transplant	1
HIV/AIDS and multicentric Castleman disease	1
Systemic lupus erythematosus	0
Drug formulation (oral vs IV), no/No. (%)	15/18 (83.3)
Completed or documented clinical outcomes, no./No. (%)	8/18 (44.4)^a^
Completion of 14 d of therapy, no./No. (%)	8/8 (100)
Clinical outcome, no./No. (%)	
Recovered from mpox infection	7/8^b^ (87.5)
Lesions or pain first started to improve, treatment day, median	4 (n = 4)
Lesions and pain fully resolved, treatment day, median	14 (n = 1)

Data are presented as No. (%) unless otherwise indicated. This subgroup was characterized by a higher degree of severe immunocompromise and superimposed infections but ultimately achieved similar rates of improvement and resolution. Full clinical resolution of mpox disease was assessed at posttreatment assessment. Although more than half of patients were lost to follow-up, all patients demonstrated clinical improvement in illness prior to discharge (though assessment was limited in those who left against medical advice).

Abbreviations: HIV, human immunodeficiency virus; IBD, inflammatory bowel disease.

^a^One patient was confirmed to have never taken tecovirimat (left against medical advice); thus, respective outcomes are excluded.

^b^One patient had refractory and persistent disease, and died outside the timeframe of this study.

## DISCUSSION

We describe 130 patients with suspected or confirmed human mpox infection prescribed tecovirimat therapy during the 2022 global outbreak, as well as the clinical course of 89 patients in this group with documented end-of-treatment outcomes. In general, patients were prescribed tecovirimat due to the lesions affecting anatomically sensitive areas, risk of immunosuppression, and/or pain. We assume that the vast majority of those who had mpox who did not receive tecovirimat experienced self-limited disease or limited access to tecovirimat, as described elsewhere [7, 8, 15, 19]. Consistent with other reports, most patients with mpox in our cohort identified as MSM and the majority also were PWH (61.5% of our cohort had HIV, which is higher than reported in most other published studies) [7, 14, 15, 35]. The higher number of PWH in our cohort is likely due to the high prevalence of PWH within New York City, as well as the structure of our tecovirimat prescribing program, which was primarily conducted through our ambulatory HIV program [36].

The clinical presentation of mpox—quantity and location of lesions—was similar among PWH and those not immunocompromised. There were no differences in the rate of recovery, hospitalization, or superimposed bacterial infection, which is consistent with another recently published cohort study [15]. Notably, 14% of our cohort required hospitalization (n = 18). and tecovirimat initiation in the inpatient setting is higher than what has been described in other series, which may be explained by the number of patients in the cohort with severe immunocompromise [7, 12, 14, 15]. Compared to the largest observational analysis of hospitalized patients to date (which had fewer immunosuppressed patients than our study and in which uncontrolled pain was the most common reason for admission), patients in our cohort primarily required admission due to superinfection of existing lesions [14, 34]. The WHO recently analyzed a large database of nearly 35 000 PWH and similarly observed that only patients who were significantly immunosuppressed were at increased risk for hospitalization, but that HIV alone was necessarily not a risk factor [14, 18, 33, 34, 37].

Consistent with the accumulating body of literature, tecovirimat was well tolerated with relatively minimal reported side effects [14, 15, 18–20, 30–32, 38]. Most patients in this cohort who received tecovirimat clinically recovered from mpox infection with no clinically significant adverse events attributed to tecovirimat. Patients in this cohort improved rapidly on treatment, similar to self-reported subjective improvement of lesions for those taking tecovirimat observed in other cohorts [14, 15, 31]. The efficacy of tecovirimat in immunocompromised patient populations was beyond the scope of this study but is an important topic for further research.

Limitations of this study include the high rate of loss to follow-up, subjective nature of prescribing, and small sample size. In addition, individuals prescribed tecovirimat beyond the timeframe of this study (1 July to 1 October 2022), including patients who were diagnosed with mpox after 1 October, are excluded from this report. Data pertaining to STI screening are limited. Although screening for concomitant STIs were recommended, adherence to recommendations was hampered by limitations intrinsic to telehealth as well an abundance of referrals from outside of our healthcare system. Consequently, we are only able to report results available in our electronic health record and by report at the time of the initial visit.

Additionally, frequent changes in the CDC EA-IND protocol increased prescribing but resulted in less strict follow-up requirements. Nearly a third of patients did not return for posttreatment assessments, consistent with other retrospective studies [15]. We hypothesize that this was likely due to rapid symptom resolution and a lack of incentive for patients without other comorbidities or established care to return solely for treatment follow-up. Additionally, due to the density of hospitals and clinics in New York City, there is a possibility that patients were hospitalized elsewhere or chose to follow up with another clinician or practice. Although difficult to measure, our approach to treatment also evolved and willingness to prescribe therapy increased over the course of the outbreak as providers became more comfortable with a less onerous prescribing process and local access to medication improved through home delivery programs.

In conclusion, in this cohort of patients prescribed tecovirimat during the 2022 mpox outbreak, nearly all patients experienced clinical recovery. However, a significantly higher level of hospitalization was seen in patients with severe immunocompromise, consistent with global reports of severe disease in patients with severe immunodeficiency. Although there has been a reversal of the public health emergency status and a significant decrease in incidence, reports continue to describe cases of mpox outside of travel [39]. Given the potential for severe infection and associated morbidity, our results support the ongoing need for prospective studies like STOMP, particularly in immunocompromised patients, and additional clinical trials for mpox therapeutic options.

## Supplementary Material

ofad552_Supplementary_Data

## Supplementary Data

Supplementary materials are available at *Open Forum Infectious Diseases* online. Consisting of data provided by the authors to benefit the reader, the posted materials are not copyedited and are the sole responsibility of the authors, so questions or comments should be addressed to the corresponding author.

## Notes

***Acknowledgments.*** The authors thank all of the colleagues and staff who were directly and indirectly involved in the care of these patients, and for their continued service and contributions, as well as Robin Scott, who aided with graphic design.

***Financial support.*** No funding was available or used for this study.

***Patient consent.*** This is a retrospective study using de-identified data; the retrospective study was approved by the ISMMS IRB. Patient consent was required for the prescribing of tecovirimat in accordance with the CDC IRB-approved EA-IND protocol.
