# Supplementary material for: Clinical Characteristics and Outcomes of Patients With Mpox Who Received Tecovirimat in a New York City Health System
Source: Open Forum Infect Dis. 2023 Nov 2;10(11):ofad552. doi: 10.1093/ofid/ofad552 (PMC10644828; doi:10.1093/ofid/ofad552)
Supplement: ofad552_Supplementary_Data [file ofad552_supplementary_data.docx]

| **Supplemental Table 1. Demographic and clinical characteristics of persons with HIV infection** | |
| --- | --- |
| **Characteristic** | **Persons with HIV Infection (n=80)** |
| **Median age (range) — yr (range)** | 39 (20-58) |
| **Male sex assigned at birth — no. (%)** | 80 (100) |
| **Sexual orientation — no. (%)** |  |
| MSM | 76 (95) |
| Heterosexual | 0 |
| Sex with men and women | 3 (3.8) |
| Other | 1 (1.3) |
| **CD4 T-cell count (cells/mL) - no (%)** |  |
| CD4 500+ | 41 (51.3) |
| CD4 200-499 | 20 (25) |
| CD4 0-200 | 11 (13.8) |
| Unknown | 8 (10) |
| Average (cells/mL) | 533 |
| Median (cells/mL) | 522 |
| **HIV viral load (copies/mL) — no. (%)** |  |
| Undetectable (<20 copies) | 39 (48.8) |
| 20-200 | 18 (22.5) |
| >200 | 15 (18.8) |
| Not reported | 8 (10) |
| Average | 28,127 |
| Median | 20 or less |
| **Clinical diagnosis of HIV/AIDS (based on total CD4 count <200 cells/mL and/or ≤13% of total lymphocytes) — no. (%)** | 13 (16.3) |
| **Reporting to be taking antiretroviral therapy — no. (%)** | 75 (93.8) |

**Supplemental Table 1. Demographic and clinical characteristics of persons with HIV infection.** Basic demographics and clinical status of HIV disease within cohort.

| **Supplemental Table 2. Reported adverse effects of tecovirimat** | |
| --- | --- |
| **Adverse Event** | **All persons (N=85)** |
| **Serious adverse event - no. (%)** | 0 |
| **No reported adverse event - no. (%)** | 78 (91.8) |
| **Any non-severe adverse effect - no. (%)** | 7 (8.2) |
| Abdominal discomfort | 3 |
| Back pain | 1 |
| Diarrhea | 1 |
| Drowsiness | 1 |
| Headache | 3 |
| Irritability | 1 |
| Nauseas | 2 |
| Steatorrhea | 1 |

**Supplemental Table 2. Reported adverse effects of tecovirimat.** This data only pertains to patients who were seen at their post-treatment follow-up appointment per CDC-IND protocol.


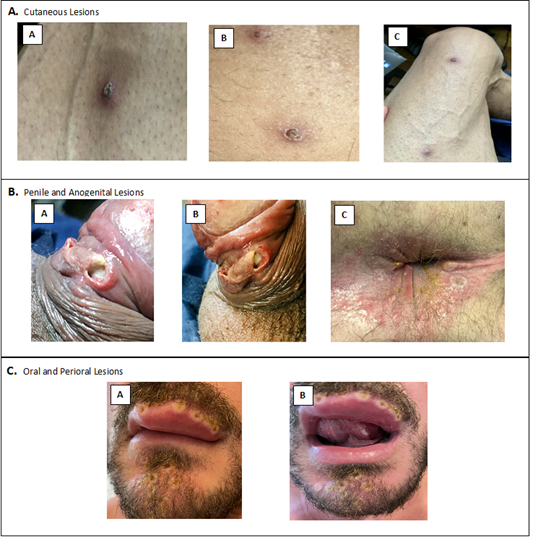


**Supplemental Figure 1. Example images of mpox lesions.** A. Cutaneous lesions (a-c); B. Penile and Anogenital lesions (a-c); C. Oral and Perioral lesions (a & b).
